# Prognostic Factors of Proteinuria Remission in Primary Membranous Nephropathy

**DOI:** 10.3390/jcm14092880

**Published:** 2025-04-22

**Authors:** Kornelia Krakowska-Jura, Anna Natalia Kler, Weronika Wajerowska, Andrzej Konieczny, Mirosław Banasik

**Affiliations:** 1 Clinical Department of Nephrology, Transplantation Medicine and Internal Diseases, Institute of Internal Diseases, Wroclaw Medical University, 50-565 Wroclaw, Poland; andrzej.konieczny@umw.edu.pl (A.K.); miroslaw.banasik@umw.edu.pl (M.B.); 2 Faculty of Medicine, Wroclaw Medical University, 50-565 Wroclaw, Poland; anna.kler@student.umw.edu.pl (A.N.K.); weronika.wajerowska@student.umw.edu.pl (W.W.)

**Keywords:** proteinuria, membranous glomerulonephritis, biomarkers

## Abstract

Primary membranous nephropathy is a leading cause of nephrotic syndrome in adults, characterized by immune complex deposition in the glomerular basement membrane. Predicting proteinuria remission is essential for guiding treatment decisions, optimizing immunosuppressive therapy, and improving renal outcomes. Traditional prognostic markers, such as anti-PLA2R antibody status and baseline proteinuria levels, offer valuable insights into disease progression. However, recent research has identified additional biomarkers that may enhance risk stratification and refine individualized treatment strategies. Serum-based markers, such as uric acid and inflammatory indices, may indicate systemic changes that impact disease progression. Urinary biomarkers, including microhematuria, α1-microglobulin, and CXCL13, have been proposed as potential predictors of disease activity and remission likelihood. Furthermore, histopathological features, such as glomerular basement membrane thickness, tubulointerstitial injury, and acute kidney injury, provide structural correlates that may inform prognosis. This review explores both established and emerging prognostic indicators across various biological domains. Understanding these predictors can aid in developing personalized therapeutic strategies, optimizing disease management, and improving patient outcomes in primary membranous nephropathy.

## 1. Introduction

Primary membranous nephropathy (PMN) remains a significant challenge in nephrology due to its unpredictable course and variable response to treatment. While spontaneous or treatment-induced remission of proteinuria is a key determinant of renal outcomes, the factors predicting remission, such as antibodies to the phospholipase A2 receptor (PLA2R) status and baseline proteinuria levels, remain incompletely understood. Contemporary studies suggest that additional factors may refine risk stratification. Accurately identifying patients at higher or lower risk of remission remains an ongoing clinical need. Recent advances suggest that additional biomarkers—spanning serum, urinary, and histopathological parameters—may offer a more comprehensive assessment of disease prognosis. By integrating these predictive markers into clinical practice, nephrologists may enhance disease monitoring, improve treatment decision-making, and ultimately optimize patient outcomes.

## 2. Primary Membranous Nephropathy: Pathophysiology and Diagnosis

Membranous nephropathy (MN) is a non-inflammatory autoimmune glomerular disease, characterized histologically by the thickening of the glomerular basement membrane (GBM) due to the deposition of immune complexes. MN can be classified as primary or secondary, with the former constituting approximately 80% of cases. The disease primarily affects adults, with a peak incidence between 50 and 60 years of age, and a male-to-female ratio of approximately 2:1 [1]. Despite a heterogeneous clinical course, 30–40% of patients achieve spontaneous remission, while 30% progress to end-stage kidney disease (ESKD) [2]. PMN is responsible for approximately 20–30% of nephrotic syndrome (NS) cases in adults [3]. The condition typically manifests with nephrotic-range proteinuria (>3.5 g/day), hypoalbuminemia, hyperlipidemia, and peripheral edema. Unlike inflammatory glomerulopathies, PMN usually presents without hematuria or significant renal dysfunction at onset. However, persistent proteinuria is associated with progressive renal impairment and increased cardiovascular risk. Identification of PLA2R as the major autoantigen, in 2009, revolutionized the understanding of PMN, with anti-PLA2R antibodies detected in 70–80% of cases [1]. Other antigens, including thrombospondin type-1-domain containing 7A (THSD7A), neural epidermal growth factor-1 (NELL-1), and Semaphorin 3B, have been implicated in PMN pathogenesis [4]. Complement activation, particularly via the membrane attack complex (MAC), contributes to podocyte injury, proteinuria, and progressive glomerular damage. Proteinuria is the hallmark clinical feature of PMN, directly resulting from podocyte injury and dysfunction of the glomerular filtration barrier. The severity of proteinuria correlates with disease activity and prognosis, with levels above 8 g/day indicating a higher risk of progression to chronic kidney disease (CKD). Persistent nephrotic-range proteinuria contributes to hypoalbuminemia, hypercoagulability, and dyslipidemia, increasing the risk of thromboembolic events and cardiovascular complications. Early and accurate diagnosis of PMN relies on serologic and histopathologic assessments. Non-invasive diagnosis relies on the detection of circulating anti-PLA2R and anti-THSD7A antibodies [2]. Complement activation markers, such as C5b-9, are often elevated and may contribute to podocyte injury [3]. In kidney biopsy, light microscopy reveals thickened glomerular capillary walls, while electron microscopy demonstrates subepithelial immune deposits. Immunofluorescence typically shows granular IgG and C3 deposits [1]. Numerous studies have confirmed the genetic causes of primary membranous nephropathy. A genome-wide association study has shown that variants in PLA2R1 and HLA-DQA1 are strongly associated with idiopathic membranous nephropathy in patients of European descent [5]. First and foremost, a strong association between membranous nephropathy and the HLA-B8DR3 haplotype was identified. Recent studies of the human genome have also revealed significant associations between the 6p21 HLA-DQA1 and 2q24 PLA2R1 loci [6]. Xie et al. have defined previously unreported loci: DQA10501 as a primary risk allele region in Europeans, DRB11501 in East Asians, and DRB1*0301 in both ethnicities [7]. The risk loci for MN may serve as an equivalent and alternative method for the non-invasive diagnosis of membranous nephropathy in the future.

## 3. Management of PMN

**Figure 1:**
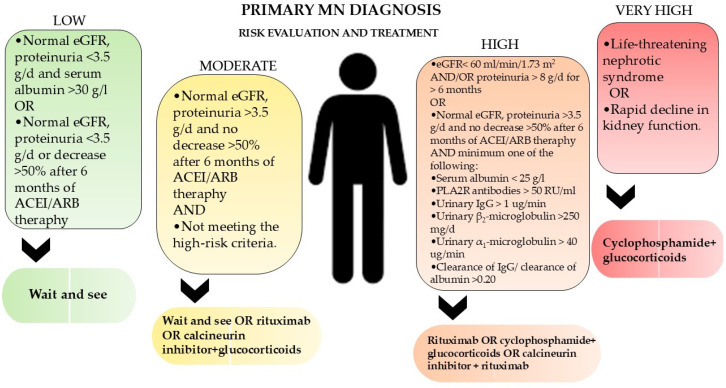
Management and treatment of membranous nephropathy according to KDIGO 2021 clinical practice guidelines for the management of glomerular diseases.

Treatment of PMN is guided by risk stratification based on proteinuria levels, anti-PLA2R titers, and renal function (Figure 1). First-line measures include renin-angiotensin system inhibitors (ACEi), lipid-lowering agents, and sodium restriction to reduce proteinuria and cardiovascular risk. In more severe cases, immunosuppressive therapy is used as a main form of treatment. This therapy includes corticosteroids, cyclophosphamide (CYC), rituximab (RTX), obinutuzumab, and calcineurin inhibitors (CNI) [3,4]. Remission in PMN is categorized as partial or complete remission based on proteinuria levels and renal function. Partial remission (PR) is defined as a reduction in proteinuria to <3.5 g/day, with at least a 50% decrease from baseline, along with stable serum creatinine levels. Complete remission (CR) is achieved when proteinuria falls below 0.3 g/day, accompanied by the normalization of serum albumin and renal function. Studies indicate that approximately 60–70% of patients treated with immunosuppressive therapy achieve partial remission, while 30–40% attain complete remission within two years.

## 4. Traditional Prognostic Factors in Proteinuria Remission in Primary Membranous Nephropathy

Prognostic Value of PLA2R Antibodies: PLA2R antibody titers have prognostic implications in PMN. Higher antibody levels at diagnosis are associated with increased proteinuria, delayed remission, and a higher risk of disease progression [8]. Conversely, declining antibody levels following treatment correlate with proteinuria reduction and clinical remission. Persistent high antibody titers indicate a need for intensified immunosuppressive therapy, while undetectable PLA2R levels suggest favorable long-term outcomes [9]. Differentiation between PLA2R-positive and PLA2R-negative PMN is critical for determining prognosis and treatment approaches. Monitoring PLA2R antibody levels helps guide treatment decisions. Patients with high antibody titers benefit from early initiation of immunosuppressive therapy. A decline in PLA2R antibody levels precedes clinical remission, allowing clinicians to assess treatment efficacy. In relapsing patients, re-emergence of PLA2R antibodies signals disease recurrence, necessitating therapeutic adjustments [10]. Ongoing research aims to refine PLA2R antibody quantification and identify additional biomarkers to complement PLA2R testing. The development of targeted therapies against PLA2R-mediated autoimmunity represents a promising avenue for future treatment.

Prognostic value of other antibodies linked to MN: Recent discoveries using laser microdissection and mass spectrometry have identified several novel antigens in PLA2R-negative membranous nephropathy, each with distinct clinical features and prognostic implications. THSD7A-associated MN accounts for approximately 2–5% of primary MN cases and is characterized by the presence of circulating anti-THSD7A autoantibodies. This subtype has been linked to a higher prevalence of malignancies compared to PLA2R-associated MN, suggesting a potential paraneoplastic mechanism in some patients. Immunohistochemically, THSD7A localizes to the podocyte membrane, and affected glomeruli show granular subepithelial immune complex deposition similar to other MN variants. EXT1/EXT2-associated MN occurs mostly in young females and is linked to autoimmune diseases, particularly lupus, with a relatively favorable prognosis. NELL1-associated MN typically affects older adults, sometimes in association with malignancy, and may present with detectable serum antibodies. Sema3B-associated MN is mainly seen in children and young adults, occasionally with a familial pattern, and shows variable treatment responses. PCDH7-associated MN affects older adults, often without autoimmune or malignant associations, and may follow a benign course with potential for spontaneous remission. These findings support a more precise, antigen-based classification of MN to better guide diagnosis and management [11].

Baseline Proteinuria Levels—Proteinuria severity at diagnosis is a strong predictor of remission. Patients with lower baseline proteinuria (<4 g/day) have a higher likelihood of achieving remission, while those with persistent nephrotic-range proteinuria (>8 g/day) are at increased risk of disease progression [8]. Persistent high proteinuria contributes to further renal damage through mechanisms such as podocyte injury, tubulointerstitial fibrosis, and increased glomerular permeability. Studies indicate that spontaneous remission occurs in a subset of patients with lower proteinuria levels, whereas higher levels typically require immunosuppressive therapy for effective management [9].

Renal Function (eGFR and Serum Creatinine): The estimated glomerular filtration rate (eGFR) and serum creatinine (sCr) concentration at diagnosis influence remission probability. Patients with preserved renal function (eGFR > 60 mL/min/1.73 m^2^) have better remission rates compared to those with impaired renal function [12,13]. A declining eGFR indicates progressive kidney damage, often making remission less likely.

Age: Age is a significant determinant in PMN progression. Studies indicate that older patients (≥60 years) present with higher sCr and are more likely to have hypertension at diagnosis [13]. However, despite worse renal function at baseline, remission rates between older and younger patients appear comparable, suggesting that early diagnosis and appropriate management can mitigate age-related risks [13]. Older patients often require careful monitoring due to comorbidities that may influence disease progression. The role of aging-related inflammation and its impact on PMN outcomes is an area of active investigation, with implications for targeted therapeutic strategies.

Hypertension: Hypertension is both a consequence and an exacerbating factor of PMN. Elevated blood pressure is associated with increased glomerular sclerosis, interstitial fibrosis, and a lower cumulative renal survival rate. Multivariate analysis identifies diastolic blood pressure as an independent risk factor for progression to ESRD [14]. Managing hypertension in PMN patients is critical for delaying renal function decline. ACEi and angiotensin receptor blockers (ARBs) have demonstrated efficacy in reducing proteinuria and preserving renal function.

Hyperlipidemia: Hyperlipidemia, commonly observed in PMN patients, correlates with proteinuria persistence. Elevated levels of total cholesterol, LDL, and non-HDL cholesterol are predictive of lower proteinuria remission rates [15]. Notably, non-HDL cholesterol exhibits the strongest correlation with disease activity, underscoring its role as a potential therapeutic target [15]. Lipid-lowering therapies, including statins, have been explored as adjunctive treatments to mitigate PMN progression.

Treatment Response to Immunosuppressive Therapy: Patients receiving immunosuppressive therapy, such as CNI, CYC, or RTX, show varying responses. Early reduction in proteinuria and PLA2R antibody levels predicts long-term remission, while treatment resistance necessitates alternative strategies [16].

Interestingly, renal function at diagnosis and type of immunosuppressive treatment did not significantly predict remission likelihood [17]. Genetic predisposition, inflammatory markers, and novel autoantibodies are emerging as potential predictors of remission. Further studies are needed to validate these factors and incorporate them into clinical practice for more refined prognostic models.

## 5. New Prognostic Markers in Proteinuria Remission in Primary Membranous Nephropathy

Serum markers: Proteinuria remission markers in PMN can be recognized among factors found in blood tests, such as level of hemoglobin, serum uric acid, neutrophil-to-lymphocyte ratio, fibrinogen–album ratio, and inflammation biomarkers.

Anemia frequently occurs in patients with PMN, typically presenting as mild to moderate normocytic anemia, although its cause of development is independent of renal tubular lesions at the diagnosis of PMN. Studies indicate that a high hemoglobin level is a positive prognostic factor for remission [18,19], and patients treated for anemia have a greater chance of a better outcome [18].

Another serum marker of remission in proteinuria is the level of uric acid (UA) in the blood. Research on its relationship with disease progression has shown that high concentrations of UA (above 335 μmol/L, as in the study conducted by Zhang et al.) are associated with a higher risk of developing chronic kidney disease, including MN [20,21]. Elevated UA levels often coincide with increased fibrinogen levels, which together serve as markers of inflammation, as well as with elevated levels of other classical markers of disease progression, such as low eGFR level and hypertension [20].

Other studies consider the role of inflammation cells and markers as MN remission prognostic factors. One of them is neutrophil-to-lymphocyte ratio, which has been studied in various diseases, and in PMN serves as an independent risk factor for proteinuria non-remission, particularly in patients with CKD stage 3–4 and 24 h proteinuria ≥ 1 g [22,23]. The two studies, which collectively involved over 600 patients with a minimum follow-up period of 12 months, demonstrate a lower rate of proteinuria remission in patients exhibiting a high neutrophil-to-lymphocyte ratio (NLR). The respective NLR cut-off values were 2.63 and 2.61.

Serum fibrinogen–albumin ratio (FAR) has been proved to serve a prognostic function in PMN as well. High FAR correlates positively with proteinuria and anti-PLA2R while being an independent risk factor for disease progression. PMN patients with low FAR have a greater chance of remission. In addition, this marker correlates with anti-PLA2R antibodies and proteinuria. Studies show that the predictive value of anti-PLA2R antibodies in PMN remission is enhanced while combined with FAR [24].

Inflammation biomarkers play a significant role in PMN remission prediction, for example, systemic immune inflammation index (SII) and pan-immune inflammation value (PIV). Those new markers are calculated with neutrophil, lymphocyte, thrombocytes, and monocyte counts in peripheral blood and are reflections of the total systemic inflammation status of the patient. Research shows that SII and PIV can act as markers for non-remission in patients with low- and moderate-risk MN, with PIV being more reliable than SII [25].

Urinary markers: Urine tests are sources of various proteinuria remission prognostic factors in MN, such as microhematuria, urinary levels of α1-microglobulin, urinary levels of CXCL13, and inflammation biomarkers.

In general, hematuria is a clinical feature of multiple kidney diseases. Microhematuria is common among PMN patients, with approximately 60% presenting with this occurrence. Studies indicate that initial microhematuria in PMN is a marker of relapse and the persistence of microhematuria correlates with disease progression, whereas its remission might improve renal outcomes [26,27].

Other studies show that urinary α1-microglobulin can act as an early and accurate predictor of PMN progression [28]. Urinary level of C-X-C motif chemokine ligand 13 (CXCL13) has been proved to predict personal response to tacrolimus and RTX proteinuria treatment, which can be used to classify patients into low-risk and high-risk groups. Cytokine biomarkers should be regarded as supplementary to currently used indicators, such as GFR, proteinuria, and anti-PLA2R antibodies. However, they can predict disease outcomes prior to treatment initiation and in early treatment stages, which distinguishes them from traditional markers [29].

Inflammation markers detected in urine are also valuable predictors of disease remission. Complement activation plays a crucial role in the pathogenesis of autoimmune glomerular diseases such as MN, which is why some studies demonstrate the potential role of urinary sC5b-9, MCP-1, and TGF-β1 as proteinuria remission prognostic factors. It has been proved that compared to proteinuria, urinary sC5b-9 is a more sensitive MN remission marker, while MCP-1 and TGF-β1 are less different in disease progression prediction than proteinuria [30].

Kidney tissue-related markers—Among prognostic markers directly associated with kidney tissue, studies mention chronic tubulointerstitial injury, glomerular basement membrane thickness, and acute kidney injury.

The mechanism of tubulointerstitial damage (TID) lesions in PMN is unclear, although studies have found TID to be a dependent risk factor of disease progression [21,31]. It is associated with high levels of 24-h urine protein, blood urea nitrogen, serum creatinine, cystatin C, β2-microglobulin, and anti-PLA2R. TID was also proved to coexist with other lesions in glomeruli samples, such as spherical sclerosis, glomerular mesangial hyperplasia, inflammatory cell infiltration, small vessel lesions, acute tubulointerstitial lesions, balloon adhesion, and focal segmental glomerulosclerosis [31].

Glomerular basement membrane thickness is a typical histopathological characteristic used in PMN diagnosis, although it has been proven that it can also be used as a prognostic marker in disease remission. A study comparing various patients’ GBM thickness showed that it might serve as an independent risk factor for progression. It has also been found that severe GBM thickness is associated with higher titers of anti- PLA2R antibodies and PLA2R antigen deposition [32].

When it comes to acute kidney injury (AKI), research shows that it may affect complete remission in PMN. The occurrence rate of AKI in PMN varies between 24.11% and 38.42% [12]. Patients with AKI have significantly lower complete remission rates than patients without AKI. The pathological changes in AKI in NM are mostly associated with acute tubular injury [33]. Other studies have found AKI to be associated with higher TID and chronicity index (CI) score. Other findings include that patients with higher levels of serum uric acid have a greater chance of developing AKI [34].

## 6. Conclusions

Primary membranous nephropathy is a leading cause of nephrotic syndrome in adults, and understanding its remission predictors is crucial for managing renal outcomes effectively. Established prognostic markers include PLA2R antibody status and baseline proteinuria levels, but emerging indicators like serum uric acid and inflammatory markers provide additional insights. High antibody levels indicate a delayed remission, whereas declining levels correlate with clinical improvement. Moreover, low baseline proteinuria, preserved renal function, and early treatment response significantly enhance remission chances. Age, hypertension, and hyperlipidemia also influence disease progression, emphasizing the need for comprehensive management strategies. Indicators found in blood tests, such as level of hemoglobin, are valuable in disease progression prognosis as well. Identifying novel biomarkers, such as the neutrophil-to-lymphocyte ratio and fibrinogen–albumin ratio, could refine risk stratification further. Urinary markers like α1-microglobulin, microhematuria, and CXCL13, alongside kidney tissue-related factors like glomerular basement membrane thickness, chronic tubulointerstitial injury, and acute kidney injury, also hold potential for predicting disease outcomes. Overall, incorporating these prognostic tools into clinical practice could optimize therapeutic approaches, ultimately improving patient prognosis in PMN. New research directions seeking additional prognostic factors may further refine the diagnostic and therapeutic process for both new and existing patients.

## Data Availability

No new data were created or analyzed in this study.

## Abbreviations

The following abbreviations are used in this manuscript:

AKI	acute kidney injury
ACEi	renin-angiotensin system inhibitor
ARB	angiotensin receptor blocker
CI	chronicity index
CKD	chronic kidney disease
CNI	calcineurin inhibitors
CR	complete remission
CXCL13	C-X-C motif chemokine ligand 13
CYC	cyclophosphamide
ESKD	end-stage kidney disease
FAR	serum fibrinogen-albumin ratio
GBM	clomerular basement membrane
eGFR	estimated glomerular filtration rate
MAC	membrane attack complex
MN	membranous nephropathy
NELL-1	neural epidermal growth factor-1
NS	nephrotic syndrome
PLA2R	phospholipase A2 receptor
PR	partial remission
RTX	rituximab
SII	systemic immune inflammation index
sCr	serum creatinine
PIV	pan-immune inflammation value
PMN	primary membranous nephropathy
THSD7A	thrombospondin type-1-domain containing 7A
TID	tubulointerstitial damage
UA	uric acid
